# Percutaneous Coronary Intervention in an 8-Month-Old Infant for Ostial Stenosis of a Reimplanted Left Main Coronary Artery

**DOI:** 10.1155/2018/2512406

**Published:** 2018-11-08

**Authors:** Amanda Cai, Courtney Kramer, Rani Bandisode, Valerian L. Fernandes

**Affiliations:** ^1^Department of Medicine, Division of Cardiology, USA; ^2^College of Medicine, Medical University of South Carolina (MUSC), Charleston, SC, USA; ^3^Department of Pediatrics, Division of Pediatric Cardiology, USA

## Abstract

Percutaneous coronary intervention (PCI) is a routine procedure undertaken in adult patients. In children, the procedure remains rare and challenging due to a multitude of factors including but not limited to complex congenital heart disease anatomy, catheter and stent to patient size mismatch, and lack of data for post-PCI antiplatelet therapy. We present a case of PCI in an 8-month-old infant with anomalous left coronary artery from pulmonary artery (ALCAPA) who developed severe ostial kinking of the left main coronary artery (LMCA) after surgical reimplantation of the anomalous coronary. A 3.5 × 8 mm Vision bare metal stent was successfully placed into the LMCA and postdilated with excellent results. Follow-up echocardiography at 6 months post-PCI demonstrated a patent stent with normal Doppler flow signals. Despite initial success, the infant developed severe heart failure and was listed for orthotopic heart transplantation at age 20 months, one year after PCI. Given the paucity of published data regarding PCI and outcomes in infants with ALCAPA after surgical reimplantation, we describe our case and present a review of the available literature.

## 1. Introduction

Anomalous left coronary artery from the pulmonary artery (ALCAPA), also known as Bland-White-Garland syndrome, is a congenital coronary anomaly comprising 0.4% of all congenital heart diseases. The hemodynamic significance of ALCAPA arises after birth, when systemic pressures exceed pulmonary pressures and myocardial ischemia occurs as a result of low perfusion pressure in the left-sided circulation territory. Furthermore, development of heterocollateral circulation from the right-sided system to preserve the left-sided perfusion can result in coronary artery steal. Definitive treatment involves reimplantation of the LMCA from the pulmonary artery into the aorta. Coronary obstruction in the reimplanted artery is a rare complication but can result in significant myocardial ischemia, infarction, and heart failure necessitating further intervention. Percutaneous coronary intervention can be used as a minimally invasive approach to correct such complications after surgical reimplantation in ALCAPA. Despite its ubiquity in the treatment of atherosclerotic coronary artery disease in adults, PCI is performed infrequently in the pediatric population with less than 100 cases reported involving the left main coronary artery (LMCA) [1]. The small vessel size of infants in particular contribute to the complexity of PCI procedures undertaken in this age group and present a host of periprocedural complications related to catheter and stent to patient size mismatch: occlusion ischemia, vessel dissection or rupture, and creation of intimal flaps which may lead to subsequent myocardial ischemia and infarction [2]. Even in uncomplicated cases, stent selection with consideration to ongoing vessel growth poses a unique challenge to PCI in the pediatric population. Given the paucity of data with regard to PCI in infants with surgically repaired ALCAPA, we describe a case with additional review of the literature.

## 2. Case Report

### 2.1. Patient Presentation

**Figure 1:**
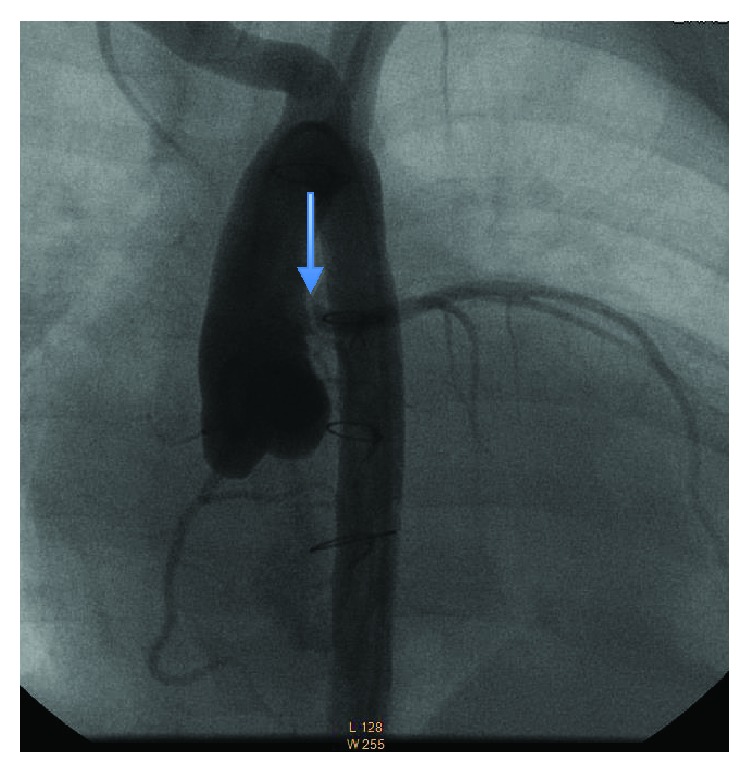
Coronary angiography demonstrating ostial LMCA stenosis.

An 8-month-old female infant had coronary reimplantation at age 3 months for ALCAPA. Postsurgical ejection fraction showed early improvement with subsequent deterioration. A diagnostic left heart catheterization performed as part of a heart transplant evaluation revealed severe ostial stenosis of the LMCA. She was referred for PCI of the left main coronary artery to relieve her heart failure and preempt transplant. Her physical exam was significant for congestive heart failure and failure to thrive. The patient's echocardiogram showed markedly depressed left ventricular function. A cardiac computed tomography angiography (CTA) and initial diagnostic nonselective root aortogram demonstrated stenosis of the LMCA at the site of ALCAPA reimplantation. Selective left coronary angiogram revealed severe kinking of the reimplanted LMCA at the ostium (Figure 1).

### 2.2. Interventional Management

**Figure 2:**
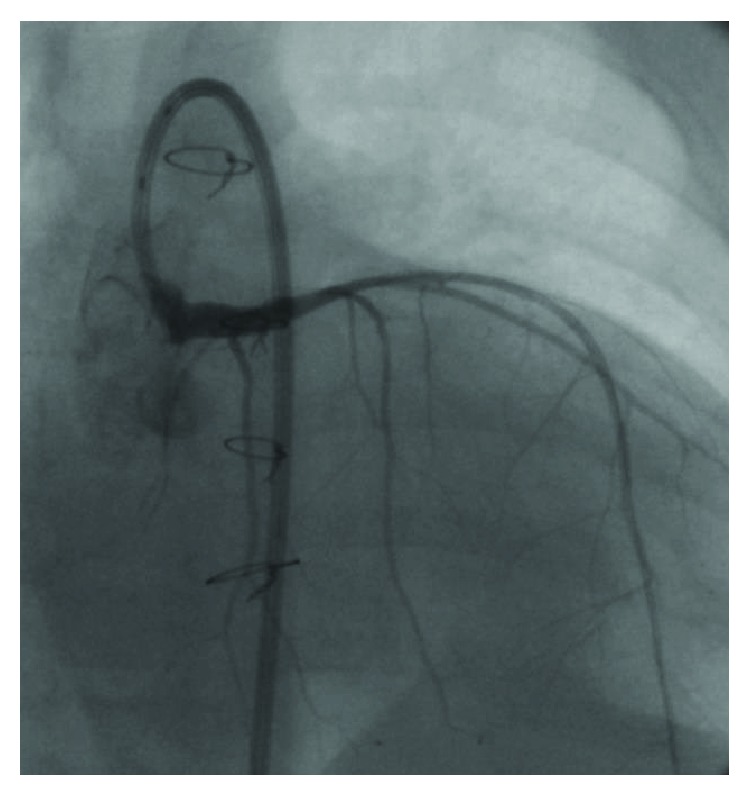
Reestablishment of LMCA perfusion after PCI.

The procedure was performed in the Pediatric Cardiology interventional suite after extensive discussion and planning with Pediatric and Adult Interventional Cardiology, Pediatric Cardiac Anesthesia, Pediatric Cardiothoracic Surgery, and Pediatric Cardiac Radiology. General anesthesia and a femoral arterial approach were utilized. An ascending aortic root angiogram was obtained in 2 planes to assess the ostial left main stenosis. Due to the small size of the infant aorta, a 6 Fr JR-4 guide was reshaped to engage the left main coronary artery. A BMW wire was used to cross the lesion. Since the infant left main coronary artery was small but was expected to grow with age, a somewhat larger (3.0 × 8 mm Vision bare metal) stent was carefully implanted in the proximal LMCA at less than nominal pressure to avoid distal dissection. A poststent angiogram showed that the stent had moved during implantation and missed the ostium with residual stenosis of the LMCA origin. Hence, a 3.5 × 8 mm Vision bare metal stent was placed into the LMCA ostium overlapping with the previous stent distally and protruding 1-2 millimeters in the aorta proximally. The ostium and aortic overhanging portion of the stent were postdilated producing proximal flaring. The final angiogram confirmed excellent stent position and normal flow (Figure 2).

### 2.3. Follow-Up

**Figure 3:**
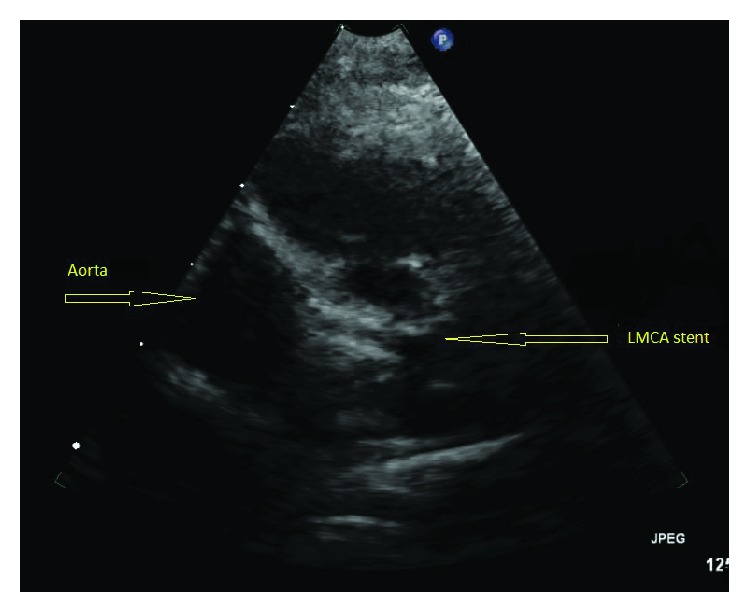
Post-PCI transthoracic echocardiogram demonstrating widely patent stent in the LMCA.

The patient tolerated the procedure well and was discharged home on dual antiplatelet therapy (DAPT) with aspirin and clopidogrel one day after PCI. She was followed in Cardiology clinic post-PCI and noted to have no change in her ventricular function. Nevertheless, she continued to do well clinically until six months post-PCI, at which time she demonstrated failure to thrive and required admission for initiation of continuous milrinone infusion. The left main stent was widely patent with normal Doppler flow signals as visualized by echocardiography (Figure 3). She was listed as status 1A for orthotopic heart transplantation (OHT) and underwent transplantation one year post-PCI, at age 20 months.

## 3. Discussion

Percutaneous coronary intervention in infants remains rare with only four prior cases reported in ALCAPA patients [3–6]. Of the four previously described cases, only one attributed the coronary obstruction to a flap of tissue from the surgical anastomosis site; the other three did not detail specific structural etiologies of coronary obstruction. Wires, balloons, and stents used in all four cases were different, as were post-PCI antiplatelet therapy regimens and follow-up intervals and modalities (Table 1).

The aforementioned variability in treatment of postsurgical coronary obstruction in ALCAPA patients highlights several challenges to performing PCI in infants. Firstly, the pathophysiology of postsurgical coronary obstruction in ALCAPA is not well understood, though it is hypothesized that traction placed on the LMCA after reimplantation in the aorta and disruption of the vaso vasorum during surgery both play a key role [7]. Nevertheless, coronary artery reimplantation remains the standard of care with alternative surgical approaches including coronary artery ligation, Takeuchi procedure (creation of an aortopulmonary window and intrapulmonary baffle), and subclavian artery anastomosis demonstrating inferior outcomes [8, 9]. Coronary artery extension techniques have been employed and proven successful in reducing traction in select populations; however, these surgical techniques are utilized mainly in palliative cases or centers were surgical experience in coronary artery reimplantation is not readily available [10–12]. Percutaneous coronary intervention thus offers an alternative to repeat operation in cases of coronary artery obstruction after reimplantation, though it may ultimately be limited by treatment of a surgical problem.

Long-term outcomes of PCI in surgically corrected ALCAPA patients are also largely unknown. Of the four previously reported cases, two reported no limitations in follow-up visits at age 6 and 7 years [5, 6], though one case required surgical intervention for recurrent mitral regurgitation in the interim. The other two only reported follow-up data within one year post-PCI [3, 4]. Instrumentation including wires, balloons, and stents used in the pediatric population are variable and in accordance with feasibility of procedure as well as preference of the interventionalist. Review of PCI across all indications in the pediatric population show that standard 0.014^″^ wires were most often employed in crossing coronary obstructions. Noncompliant coronary balloons and stents that approximated the adjacent unaffected vessel diameter were most often selected [13, 14]. Data extrapolated from a retrospective review of 33 infant stent implantations across all indications, over four years at a single institution by Stanfill et al., show that 73% of implanted stents required at least one redilation [14]. Stent thrombosis has been reported in up to 3% of pediatric coronary PCI cases and up to 7% of pediatric noncoronary PCI cases [14, 15]. Nevertheless, standardized antiplatelet therapy regimens and durations have not yet been established at present.

## 4. Conclusion

Percutaneous coronary intervention remains rare and challenging in infants. Close collaboration with Adult and Pediatric Interventional Cardiology is vital to the success of these procedures, thereby making them less challenging. Percutaneous coronary intervention is a feasible option in infants who develop coronary obstruction after surgical reimplantation for ALCAPA.

## Figures and Tables

**Table 1:** Prior reported cases of PCI in infants with ALCAPA.

Study	Patient age	Etiology of coronary obstruction	Location of coronary obstruction	Catheter used	Wire used	Balloon used	Stent used	Antiplatelet therapy	Follow-up interval and modality
Chrysant 2005	3 months	Tissue flap from surgical anastomotic site	70% mid LMCA	—	—	3.0 × 10 mm Maverick	2.25 × 8 mm Sonic Hepacoat	Aspirin (1/4 tablet)	Echo at 2 and 6 months, CTA at 6 months
Hallbergson 2015	5 weeks	Stenosis one month postoperatively	Near-total occlusion of LMCA	4 Fr Judkins right coronary	0.014^″^ intermediate support	—	2.5 × 8 mm MiniVision	Aspirin 2-4 mg/kg/day and clopidogrel 0.2-0.4 mg/kg/day	Angiography at 3 months
Kaichi 2011	9 months	Stenosis one month postoperatively	Severe stenosis of LMCA	4.2 Fr Judkins left, 15 mm	6 Fr internal mammary artery guiding, 0.014^″^ ACS Hi-Torque balance middle weight	2.5-20 mm over the wire Maverick PTCA for predilation, 3.5–12 mm monorail system Quantum Maverick PTCA for postdilation	Sirolimus-eluting Cypher	Aspirin 2 mg/kg/day for life, ticlopidine 4-5 mg/kg/day × 6 months	Angiography at 4, 12, and 67 months
Paech 2015	6 months	Stenosis 29 days postoperatively	90% LMCA	4 Fr left coronary, 1.5 modified supertorque	0.014^″^ coronary guide	Maverick Bloomington × 12 mm	Prometheus AL 3.0 × 8 mm	Acetylsalicylic acid and clopidogrel followed by acetylsalicylic acid only after platelet function testing	Angiography at 10 days and 3 and 8 months
